# Supplementary material for: The Effect of Sheep and Cow Milk Supplementation of a Low Calcium Diet on the Distribution of Macro and Trace Minerals in the Organs of Weanling Rats
Source: Nutrients. 2020 Feb 25;12(3):594. doi: 10.3390/nu12030594 (PMC7146164; doi:10.3390/nu12030594)
Supplement: Supplementary file 1 [file nutrients-12-00594-s001.pdf]

Table S1: ICP-MS detection limits

Sample	Milk	Soft tissues	Food	Serum
Unit / Element	[µg/kg]	[mg/kg]	[mg/kg]	[ng/ml]
Ag	3.00	4.00 [b]	10.0 [b]	*
As	3.00	4.00 [b]	10.0 [b]	*
Ba	3.00	40.0 [b]	10.0 [b]	*
Ca	2.50 [a]	28.0	10.0	1.00 [c]
Cd	0.50	2.00 [b]	2.00 [b]	*
Cs	3.00	8.00 [b]	0.10	1.00
Cu	5.00	0.30	20.0 [b]	20.0
Cu	3.00	2.00 [b]	10.0 [b]	*
Fe	10.0	0.50	0.40	20.0
K	2.50 [a]	8.00	10.0	500
Mg	2.50 [a]	4.00	10.0	100
Mn	3.00	0.10	10.0 [b]	*
Na	2.50 [a]	4.00	10.0	
Ni	3.00	0.20	10.0 [b]	*
P	2.50 [a]	8.00	10.0	1.00 [c]
Pb	5.00	0.80 [b]	2.00 [b]	*
Rb	3.00	8.00 [b]	0.10	5.00
Se	2.50	4.00 [b]	10.0 [b]	1.00
Sr	3.00	0.20	10.0 [b]	*
Zn	0.05 [a]	0.80	2.00 [b]	100

* Element not tested for in these samples, [a] [mg/kg], [b] [µg/kg], [c] [µg/ml]

Table S2: Sheep milk, cow milk, modified-AIN-93M diet, and Low Ca/P modified-AIN-93M diet mineral compositions as determined by ICP-MS^

Diet component	Basal		Milk	
Element	Modified-AIN-93M [μg/kg]	Low Ca/P modified-AIN-93M [μg/kg]	Sheep [μg/kg]	Cow [μg/kg]
Al *	8.32	9.68	1.72	BDL
Ba	BDL	BDL	741	167
Ca #	2.85	2.17	1.70	1.25
Ce	14.7	12.2	BDL	BDL
Co	113	97.0	164	2.05
Cr *	1.38	1.25	BDL	BDL
Cs	14.0	14.2	25.5	1.13
Cu *	5.60	4.72	0.34	0.04
Er	1.24	1.22	BDL	BDL
Fe *	44.7	41.8	1.40	0.26
K #	6.78	6.75	1.11	1.54
La	10.4	9.26	BDL	BDL
Li	105	103	8.88	BDL
Mg *	557	564	151	108
Mn *	9.87	8.88	0.13	0.02
Mo	137	107	BDL	BDL
Na #	4.65	4.54	0.90	0.005
Nd	8.13	7.44	BDL	BDL
Ni	527	494	3.63	BDL
P #	2.12	1.72	1.42	1.06
Pb	BDL	BDL	20.5	BDL
Rb *	2.72	2.71	1.80	1.92
Sr *	1.58	1.27	1.52	0.53
U	9.07	6.15	BDL	BDL
V	445	421	BDL	BDL
Y	19.3	18.3	BDL	BDL
Zn *	39.0	36.7	5.26	4.28

^BDL = mineral present bellow detection limit (Table S1).* [mg/kg]. # [g/kg].

Table S3: Summary of correlations between mineral intake and macro and trace mineral concentrations in the organs of rats consuming milk diets ^

**Mineral type**															
		**Non-essential minerals**					**Macro and trace minerals**								
**Intake per day**		**Cs**	**Pb**	**Rb**	**Sr**	**V**	**Ca**	**Cu**	**Fe**	**Mg**	**Mn**	**Mo**	**Na**	**P**	**Zn**
**Organ**	**Concentration**														
Spleen	Co	-0.224	-0.147	**-0.592****	-0.147	**0.696****	**-0.628****	**0.661****	.279*	-0.309*	**0.696****	**0.688****	0.253	**-0.644****	**0.506****
Kidney		0.311*	**0.690****	**0.685****	**0.685****	**-0.600****	**0.767****	**-0.541****	-0.172	**0.460****	**-0.585****	**-0.583****	-0.027	**0.774****	-0.257
Liver	Cu	0.184	-0.241	0.185	0.284*	**-0.447****	0.223	**-0.422****	-0.178	**-0.456****	**-0.456****	**-0.459****	-0.239	0.229	**-0.340***
Liver		-0.078	-0.145	-0.316*	**-0.385****	**0.538****	**-0.372****	**0.513****	0.315*	**0.540****	**0.540****	**0.515****	0.340*	**-0.378****	**0.509****
Spleen	Fe	-0.331*	-0.572*	**-0.530****	-0.572*	**0.603****	**-0.662****	**0.529****	0.080	**-0.372****	**0.597****	**0.596****	0.067	**-0.677****	**0.353****
Liver	Mn	0.225	0.171	0.259	**0.360****	**-0.568****	0.320*	**-0.506****	-0.169	**-0.579****	**-0.579****	**-0.584****	-0.276*	0.332*	**-0.463****
Liver	Mo	0.280*	0.551*	**0.431****	**0.520****	**-0.547****	0.525**	**-0.453****	-0.104	**-0.543****	**-0.543****	**-0.543****	-0.132	**0.529****	**-0.355****
Liver	Zn	0.218	0.308	**0.415****	**0.446****	**-0.511****	**0.446****	**-0.468****	-0.144	**-0.519****	**-0.519****	**-0.521****	-0.185	**0.458****	**-0.360****

^ Reported as Spearman rank-order correlation coefficients. * Indicates a significant correlation at $p < 0.05$. ** Indicates a significant correlation at $p < 0.01$.

Table S4: Correlations between the intake of minerals (macro trace and non-essential) and the concentrations of non-essential minerals in the organs of rats ^

Mineral type		Non-essential minerals					Macro and trace minerals									
Intake per day																
**Concentration**	**Organ**	**Cs**	**Pb**	**Rb**	**Sr**	**V**	**Ca**	**Co**	**Cu**	**Fe**	**K**	**Mg**	**Mn**	**Mo**	**P**	**Zn**
As	Kidney	0.041	0.325	-0.289*	-0.112	0.158	-0.109	0.156	0.251	0.201	-0.165	-0.134	0.174	0.174	-0.115	0.117
	Liver	-0.024	-0.610*	-0.352*	-0.280*	0.330*	-0.354*	-0.004	0.328*	0.217	0.001	0.331*	0.331*	0.311*	-0.353*	0.258
	Spleen	-0.174	0.055	-0.526**	**-0.526****	**0.616****	**-0.553****	-0.113	**0.585****	0.266	0.017	-0.280*	**0.607****	**0.595****	**-0.567****	**0.469****
Cs	Brain	0.334*	0.409	-0.038	0.409	-0.187	0.223	0.363*	-0.09	0.192	-0.226	0.071	-0.185	-0.191	0.217	-0.161
	Kidney	**0.702****	0.143	-0.110	**0.562****	**-0.422****	0.338*	0.763**	-0.109	**0.389****	**-0.529****	0.053	**-0.380****	**-0.412****	0.334*	-0.269*
	Liver	**0.606****	**0.676****	0.151	**0.595****	**-0.462****	**0.466****	0.622**	-0.255	0.170	-0.353*	**-0.424****	**-0.424****	**-0.444****	0.460**	-0.304*
	Spleen	**0.796****	-0.247	-0.118	-0.118	-0.330*	0.246	0.792**	-0.109	**0.474****	**-0.484****	0.097	-0.307*	-0.355**	0.242	-0.226
	Serum	**0.700****		-0.006					-0.030	**0.447****	**-0.312***	0.197	-0.224		**0.298***	
Rb	Brain	0.193	0.556*	**0.403****	0.556*	**-0.568****	**0.516****	0.125	**-0.497****	-0.136	-0.090	0.200	**-0.571****	**-0.581****	**0.526****	**-0.424****
	Kidney	0.130	0.489*	**0.747****	**0.602****	**-0.659****	**0.701****	-0.043	**-0.689****	-0.362**	0.185	**0.404****	**-0.670****	**-0.665****	**0.723****	**-0.345****
	Liver	0.070	0.589*	**0.524****	**0.462****	**-0.551****	**0.542****	0.024	**-0.514****	-0.272*	0.036	**-0.551****	**-0.551****	**-0.537****	**0.549****	**-0.358****
	Spleen	0.299*	0.275	**0.634****	**0.634****	**-0.649****	**0.680****	0.127	**-0.652****	-0.189	0.096	**0.420****	**-0.662****	**-0.684****	**0.691****	**-0.324***
	Serum	0.231		**0.699****					**-0.442****	-0.100	0.273	**0.525****	**-0.462****		**0.771****	
Sr	Brain	0.078	0.258	-0.279	0.258	-0.115	-0.172	0.092	-0.115	0.040	-0.348*	-0.291*	-0.143	-0.156	-0.161	-0.207
	Kidney	0.290*	-0.082	**-0.350****	0.018	-0.169	-0.166	**0.350****	-0.016	0.160	**-0.521****	-0.294*	-0.171	-0.205	-0.149	-0.236
	Liver	0.179	-0.007	-0.115	0.048	-0.290*	-0.036	0.165	-0.242	-0.047	**-0.399****	-0.295*	-0.295*	-0.316*	-0.023	-0.252
	Spleen	**0.468****	-0.201	-0.227	-0.227	-0.152	-0.091	**0.396****	-0.057	0.292*	**-0.399****	-0.081	-0.167	-0.228	-0.075	-0.16

^ Reported as Spearman rank-order correlation coefficients. * Indicates a significant correlation at $p < 0.05$. ** indicates a significant correlation at a $p < 0.01$
